# Cardiac surgery in the time of the coronavirus

**DOI:** 10.1111/jocs.14580

**Published:** 2020-04-25

**Authors:** Daniel P. Fudulu, Gianni D. Angelini

**Affiliations:** ^1^ Department of Cardiac Surgery Bristol Heart Institute Bristol UK

**Keywords:** cardiac surgery, coronavirus, Covid‐19 disease, pandemic, SARS Covid‐19

## Abstract

The current Covid‐19 pandemic is a significant global health threat. The outbreak has profoundly affected all healthcare professionals, including heart surgeons. To adapt to these exceptional circumstances, cardiac surgeons had to change their practice significantly. We herein discuss the challenges and broad implications of the Covid‐19 pandemic from the perspective of the heart surgeons.

As of April 10, 2020, there are a total of 1 521 252 confirmed cases of Coronavirus Disease 2019 (Covid‐19), including 92 798 deaths reported to WHO.^1^ The pandemic has affected more than 190 countries around the world.^2^ Described initially as a pneumonia of unknown cause^3^ that was detected in Wuhan City, Hubei Province of China, in December 2019, the SARS Covid‐19 virus outbreak is now a global health threat with profound social and professional implications. Due to its effective transmission, more than a third of the global population is currently in lockdown as part of a mitigation strategy that aims to reduce the capacity of the virus to kill by increasing the ability of the health services to cope with the surge in cases.^4^

Some of us might have thought that cardiac surgeons will not be in the Covid‐19 battle frontline. However, due to the magnitude of this pandemic, we are all in this together. Therefore, many cardiac surgeons are now facing an abrupt change to their daily practice and even in their speciality theme. Somehow, we now have to forget that we operate on hearts for a living. We now have to understand that we are first doctors then surgeons. At the peak of this crisis, many cardiac surgeons find themselves working in Covid‐19 critical care units or wards, and some volunteer to cover critical care nurse roles. It is an apocalyptic scenario that no one would have imagined a few months ago. Identifying ourselves as Covid‐19 doctors rather than cardiac surgeons can be very satisfying and meaningful. However, this comes with the anxiety of contracting the virus and potentially spreading it to our families. Sadly, doctors, including cardiac surgeons^5^ died from the coronavirus, and many others will likely lose the battle with the virus in the future.

Furthermore, the lack of adequate personal protective equipment (PPE) that was reported in many parts of the world, staff absences due to sickness or isolation can further exacerbate this anxiety and work pressure. The current lack of a reliable antibody test for medical staff does not provide the certainty that we are immune to the virus and provides no reassurance when we are sent in the frontline. Some of us might feel strong and with no risk factors, thus capable of mounting an adequate response. While the statistics show that the mortality is higher in older patients or with underlying conditions,^6^ it is worrying that we do not fully understand the immune susceptibility to develop the severe form of the disease that continues to be reported in some young and fit individuals. As healthcare workers, we are likely to be exposed to a higher viral load that can be associated with developing more severe forms of the disease.^7^

We are now part of a single team in the face of a viral tsunami. This can be very challenging and will put to the test our team player abilities. However, due to the unique leadership skills, stamina and determination within our speciality, we can rightly consider cardiac surgeons as elite troops working in exceptional circumstances.

Hospitals throughout the world had to increase their critical care bed capacity manyfold, and most of the elective cardiac surgery operations have now been postponed. In a very short time, large field hospitals are being built in the conference venues where we used to present our scientific work. Our cardiac theatre rooms are being transformed into critical care wards. Amid these unprecedented, step‐wise escalation plans,^8^, ^9^ we have to make difficult decisions about which patients we consider urgent? Most cardiac surgery units limit surgery to cases such as aortic dissection in the young patient, emergency coronary artery by‐pass not amenable to percutaneous coronary intervention or valve surgery not amendable to transcatheter aortic valve replacement. While some scenarios can be straightforward, in other cases, it is hard to ascertain which patients can be deferred and for how long? Moreover, we also have to weigh the risk of inpatient Covid‐19 infection against the risk of delaying the surgery.

To achieve a very reduced length of stay, we are now diverting a significant proportion of the urgent cases to interventional cardiology. However, how optimal are the long‐term outcomes of these percutaneous cases that were meant to be treated surgically? Once we suppress this pandemic and we return to full capacity, how are we going to deal with this considerable pool of patients with delayed procedures? This is likely to require a significant effort and planning and will likely result in increased collateral mortality.

A new challenge is operating on patients that are Covid‐19 positive or suspected as high risk for the disease. Therefore, to mitigate the infectious risk, we must cope with wearing special PPE during cardiac procedures and adhere to specific theatre protocols. We also have to respect designated hospital zones that aim to limit inpatient transfers to contaminated areas and be vigilant to assess and screen patients for Covid‐19 before transfer from peripheral hospitals. Covid‐19 outbreak has completely reshaped the way we do cardiac surgery in a matter of months.

Undoubtedly, this pandemic will be a catalyst for the rapid development and retainment of telemedicine. One example is that most of our follow‐up clinics are now run by video or telephone call. Running these clinics might be challenging since most of us are used to a physical, patient consultation. Similarly, multidisciplinary meetings and mortality and morbidity meetings are set up in a virtual space.

The volume of operating has reduced dramatically and is now limited to specific pathologies. Some cardiac surgeons might deskill during this process. Furthermore, most of the cardiothoracic training programme are now put on hold throughout the world, and there is no access to scientific conferences or exams. Once the Covid‐19 dust settles, cardiac surgeons will have to regain this lost ground.

We are dealing with a new disease in our cardiac surgical patients, and we have no understanding of it. There is no research into short term and long term outcomes of patients undergoing cardiac surgery that are Covid‐19 positive. Several studies are underway for the surgical population in general,^10^ and likely more studies are warranted in the cardiac surgery subgroup. However, we are likely dealing with a very vulnerable patient population due to the underlying cardiovascular disease that is associated with high mortality in Covid‐19 disease.^11^

Patient care should always be our primary focus; however, we also have to be aware of the current economic shutdown that will affect the jobs in our speciality and the resources available to treat our patients.

At the time of writing, the future is uncertain, and we have no clear exit strategy. Social distancing is a short term and effective solution to increasing our critical care bed capacity and to reducing the spread of the virus.^4^, ^6^ After we flatten the curve, when and how do we relax these restrictive measures to avoid a second wave of infection is unknown. Certainly lifting such restrictions prematurely could result in the so‐called “double‐humped curves” we have seen in the H1N1 (Spanish flu) pandemic 1918.^12^, ^13^ In 2020, we are in a better position, and we can use sophisticated epidemiological modelling to inform policy decisions.^4^ There is no consensus on the best therapeutic strategy for Covid‐19 disease, and the current treatment is mainly supportive.

Nevertheless, we know how to develop vaccines and have many pharmacological strategies in our armamentarium. There is an ongoing research effort underway to evaluate various pharmacological agents including antiviral medication, chloroquine, Chinese medicine products, monoclonal antibodies, and intravenous hyperimmune globulin from recovered persons.^6^ There is a hope for the development of a vaccine that could be crucial in the suppression of the outbreak. Two vaccines are in clinical evaluation and 60 more in preclinical evaluation phase.^14^ However, this race is estimated to take at least 12 to 18 months.^15^

For now, we might have to live and work in the Covid‐19 pandemic. By working together and remaining resilient, we will return to our normal lives and to our speciality—cardiac surgery where lots of work will be expecting for us.

## AUTHOR CONTRIBUTIONS

DPF and GDA: (a) Substantial contributions to research design, or the acquisition, analysis or interpretation of data; (b) drafting the paper or revising it critically; and (c) approval of the submitted and final versions.
